# Clinical outcome and hemodynamic behavior of the Labcor Dokimos Plus aortic valve

**DOI:** 10.1186/s13019-016-0561-5

**Published:** 2016-11-29

**Authors:** Torsten Christ, Konstantin Zhigalov, Wolfgang Konertz, Sebastian Holinski

**Affiliations:** Department of Cardiovascular Surgery, Charité - Universitätsmedizin Berlin, Charitéplatz 1, 10117 Berlin, Germany

**Keywords:** Stented aortic valve replacement, Biological prosthesis, Valve replacement, Echocardiography

## Abstract

**Background:**

The Labcor Dokimos Plus (LDP) is a stented externally mounted pericardial aortic bioprosthesis, which was recently introduced in Europe. Aims of the study are evaluation of operative and postoperative results as well as hemodynamic performance.

**Methods:**

One hundred consecutive patients with a mean age of 65.9 ± 10.7 years (range 35–87) and a mean EuroSCORE II of 3.1 ± 3.9 (range 0.67–24.5) underwent aortic valve replacement with the LDP. Mean valve-size was 25.2 ± 1.7 mm. Concomitant procedures were performed in 34% of the cases. Postoperative clinical data were analyzed and hemodynamic performance of the prostheses was evaluated by transthoracic echocardiography. Clinical follow-up was 100%, echocardiographic follow-up was 93% complete.

**Results:**

Intraoperatively no peculiarities occurred. Mean cross clamp times for isolated and complex procedures were 74.5 ± 20.0 min and 103.7 ± 37.1 min, respectively. Patients were extubated after a mean of 9.4 ± 15.8 h. There were no perioperative strokes. Bleeding events occurred in 4 patients. 30-day-mortality was 2%. One case of early endocarditis occurred. Echocardiography showed maximum and mean pressure gradients of 18.1 ± 6.4 and 9.6 ± 3.7 mmHg, respectively. Correspondingly to valve sizes 21, 23, 25 and 27 mm, mean pressure gradients were 17.3, 9.5, 8.5 and 10.2 mmHg, effective orifice areas were 1.92, 1.79, 2.0, 2.16 cm^2^ and indexed effective orifice areas were 1.08, 0.95, 0.99 and 1.01 cm^2^/m^2^, respectively. No relevant regurgitations occurred.

**Conclusions:**

The LDP showed operatively no peculiarities and a satisfactory clinical outcome with low perioperative morbidity and mortality. The hemodynamic performance of the implanted valve sizes was satisfactory.

## Background

Recently, a new bovine pericardial stented bioprosthesis for the aortic position, the Labcor Dokimos plus (LDP), became available in Europe. The design features are a low profile stent with externally mounted leaflets [1]. Yet, no contemporary data about clinical outcome and hemodynamic performance are available. We report about our perioperative experience with this substitute, the early clinical outcome and hemodynamic performance.

## Methods

### Patients

From October 2013 to February 2015 100 consecutive patients underwent aortic valve replacement with LDP prostheses, while a total of 358 patients received an aortic valve replacement at our institution. The decision to implant the bioprosthesis was made according to the actual guidelines [2, 3]. Baseline preoperative characteristics are displayed in Table 1.

**Table 1:** Baseline characteristics and risk stratification

Characteristic	Number
Number of patients (*n*)	100
• Age in years ± standard deviation	65.9 ± 10.7
Gender	
• Male (*n*)	77
• Female (*n*)	23
Body surface area ± standard deviation (m^2^)	2.0 ± 0.2
Aortic valve lesion	
• Stenosis (*n*)	55
• Regurgitation (*n*)	25
• Mixed lesion (*n*)	20
• Active endocarditis (*n*)	9
Predominant cardiac rhythm	
• Sinus rhythm (*n*)	73
• Atrial fibrillation (*n*)	27
Concomitant disease	
• Coronary artery disease (*n*)	38
• Arterial hypertension (*n*)	67
• Pulmonary hypertension (*n*)	7
• Renal dysfunction (*n*)	32
• Diabetes mellitus (*n*)	22
• Obesity (*n*)	30
NYHA class (Mean ± standard deviation)	2.6 ± 0.7
EuroSCORE II	3.1 ± 3.9 Range: 0.7-24.5
• Isolated aortic valve replacement	2.0 ± 1.7 Range 0.7-8.2
• Complex procedures	5.9 ± 3.8 Range 0.7-24.5

### Prosthesis

**Fig. 1:**
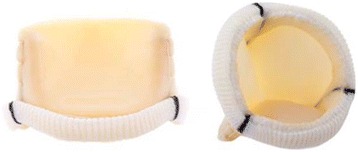
Lateral view and front view of the Labcor Dokimos Plus

The LDP prosthesis, manufactured in Labcor Laboratories, Belo Horizonte, Brazil is a CE-marked stented bovine pericardial bioprosthesis and available in sizes from 19 to 27 mm (Fig. 1). Special features of this prosthesis are a low profile, an acetal copolymer stent covered with polyester, externally mounted pre-molded leaflets fixed with glutaraldehyde at zero pressure as well as a so called Reducer® anti-calcification treatment.

### Surgical technique

A right upper hemisternotomy in the 4th intercostal space was performed for isolated aortic valve replacement and full sternotomy for combined procedures. Standard cannulation of the ascending aorta and the right atrium was performed in all cases except in two patients with additional tricuspid valve repair. In these cases bicaval cannulation was performed. Usually normothermic perfusion was used. However, in complex cases with impaired ventricular function mild hypothermia (32–34 °C) was applied. After clamping of the aorta, intermittent antegrade blood cardioplegia according to Calafiore was performed. The ascending aorta was transversely opened 1–2 cm above the commissures for half of its circumference. After resection of the diseased valve and thorough annular decalcification, sizing with ball-sizers and LDP-sizers was performed. The appropriate prosthesis was implanted with 12–20 horizontal felt-armed mattress sutures. The prosthesis was positioned either supra-annularly or intra-annularly depending on the distance between the aortic annulus and the coronary ostia, the position of the coronary ostia, calcifications in the coronary sinus and the size of the annulus and the sinus coronarius. The intra-annular position was chosen in patients with tubular sinuses, possible coronary obstruction by the bioprosthesis and in patients with aortic annuli above 29 mm. In smaller annuli (≤21 mm), stentless valves were implanted (according to institutional guidelines), what represents a selection bias for this study. Mitral valve procedures (with or without left atrial ablation), distal coronary anastomoses and aortic annular enlargement were performed before implantation of the LDP. Tricuspid procedures were performed on the beating heart after the aortic valve replacement. Aortic annular enlargement was done using the Manouguian technique using a patch of bovine pericardium to reconstruct the extended aortotomy into the non-coronary cusp and the subaortic curtain. The function of the prosthesis was controlled by trans-esophageal echocardiography.

### Clinical follow-up

After approval by the local Ethics Committee pre-, intra- and early postoperative (until discharge) data were prospectively collected. Hemodynamic performance was evaluated using transthoracic echocardiography at discharge. It was performed with a GE Vivid 7 Dimension (General Electric, Fairfield, Connecticut, USA) to check morphology and function of the implanted prostheses. Two-dimensional and Doppler transthoracic echocardiography was performed. Mean values for each measurement were derived from three beats in sinus rhythm, and five beats in those in non-sinus rhythm. Transaortic flow velocities were assessed by continuous–wave Doppler, while flow velocities in the left ventricular outflow tract were assessed by pulsed–wave Doppler. Pressure gradients were calculated using the Bernoulli equation. The effective aortic valve orifice area (EOA) was calculated with the continuity equation and indexed by the body surface area of the patient (EOAI).

### Statistics

All data were prospectively collected and analyzed with SPSS Statistics version 22.0.0 (SPSS Inc., Chicago, Illinois). Descriptive statistics are reported as the mean ± standard deviation for continuous variables and as absolute frequencies and percentages for categorical variables.

## Results

Operative details are presented in Table 2. Smaller valve sizes (≤21 mm) were implanted rarely, due to institutional guidelines to implant stentless valves in these cases. Intra-annularly implantation of the LDP was performed due to a wide aortic annulus (>29 mm, *n* = 23), possible coronary obstruction (*n* = 18) and calcification or anatomical anomalies of the Valsalva sinuses (*n* = 16). Supra-annularly implantation was performed in the rest of the patients. No intraoperative complications occurred and intraoperative mortality was 0%. Patients were extubated after a mean of 9.4 ± 15.8 h. Three patients developed a low cardiac output syndrome postoperatively and therefore received extracorporeal life support (ECLS) within the first 24 h after the operation. These patients had undergone complex combined procedures and suffered preoperatively from an impaired left ventricular function with a left ventricular ejection fraction ≤ 35%. ECLS could be weaned in two patients at the 4th and 5th day postoperatively, respectively. However, both patients died due to intractable ventricular fibrillation and septic multi-organ failure at the 9th and 44th postoperative day, respectively. The third patient died at the 6th postoperative day due to multi-organ failure. No other fatalities occurred. Hence, in patients with complex procedures the 30-day mortality was 5.9% and the hospital mortality 8.8%. For patients undergoing isolated valve replacement the 30-day mortality was 0%.

**Table 2:** Operative characteristics

Procedure	Number
Isolated aortic valve replacement (*n*)	66
Combined procedures (*n*)	34
• Coronary artery bypass grafting (*n*)	21
• Mitral Valve Replacement (*n*)	5
• Mitral Valve Repair (*n*)	3
• Left atrial ablation (*n*)	3
• Ascending Aorta Replacement (*n*)	2
• Tricuspid Valve Reconstruction (*n*)	2
• Aortic Annular Enlargement (*n*)	2
Implanted valve sizes	
• 21 mm (*n*)	3
• 23 mm (*n*)	20
• 25 mm (*n*)	41
• 27 mm (*n*)	36
Technique of implantation	
• Supra-annularly (*n*)	40
• Intra-annularly (*n*)	60
Duration of procedure (min)	212.4 ± 57.7
• Isolated procedures (min)	189.2 ± 36.3
• Combined procedures (min)	257.2 ± 65.4
Cardiopulmonary bypass time (min)	113.6 ± 40.6
• Isolated procedures (min)	96.6 ± 25.3
• Combined procedures (min)	140.2 ± 45.7
Aortic cross clamp time (min)	84.8 ± 30.0
• Isolated procedures (min)	74.5 ± 20.0
• Combined procedures (min)	103.7 ± 37.1

Postoperative complications included re-exploration for bleeding, which had to be performed in four patients. Furthermore, eight patients developed acute renal insufficiency and required temporary dialysis. Insertion of a permanent pacemaker became necessary in six patients. There were no strokes or deep sternal wound infections. One case of early postoperative endocarditis occurred, which led to a successful secondary valve replacement at the 30th postoperative day. Patients were discharged after a mean of 10.5 ± 6.9 days.

Echocardiography was analyzed for 93% of the cases. Excluded were data of 7 patients, due to insufficient conditions early postoperatively (*n* = 4), death (*n* = 2; patients with ECLS, who died on the 6th and 9th day postoperatively) and endocarditis (n = 1). Maximum and mean prosthetic pressure gradients at discharge were 18.1 ± 6.4 and 9.6 ± 3.7 mmHg, respectively. Mean EOA and mean EOAI were 2.01 ± 0.52 cm^2^ and 0.99 ± 0.25 cm^2^/m^2^, respectively. Two cases of severe patient-prosthesis mismatch (EOAI < 0.65 cm^2^/m^2^) were observed (mean body mass index in these patients was 33.3). Twenty-one cases of moderate prosthesis-mismatch (EOAI > 0.65 cm^2^/m^2^ and < 0.85 cm^2^/m^2^) were observed. No relevant central or para-valvular regurgitation was evident. No structural or nonstructural valve dysfunctions and no valve thrombosis could be observed. Table 3 provides the detailed hemodynamic data according to the different valve sizes.

**Table 3:** Echocardiographic results according to labeled valve sizes

Valve size (mm)	21	23	25	27
Number	3	18	39	33
Mean Pressure Gradient in mmHg	17.3 ± 6.7	9.6 ± 3.0	8.5 ± 3.1	10.2 ± 3.6
Maximum Pressure gradient in mmHg	29.7 ± 12.1	19.3 ± 4.9	16.5 ± 5.8	19.1 ± 5.8
Effective Orifice Area in cm^2^	1.92 ± 0.44	1.79 ± 0.36	2.0 ± 0.6	2.16 ± 0.47
Indexed Effective Orifice Area in cm^2^/m^2^	1.08 ± 0.33	0.95 ± 0.18	0.99 ± 0.29	1.01 ± 0.24

## Discussion

Conventional aortic valve replacement is still a gold standard for patients with relevant aortic valve disease without excessive risk profile. Biological substitutes are recommended for patients older than 65 years or those with contraindications to systemic anticoagulation [2, 3]. Stented biological substitutes are easy to implant and show acceptable hemodynamic performances. However, there is still the necessity to improve these valves concerning hemodynamic properties and clinical performance as well as durability. The LDP was launched in Europe in 2013. It´s innovative design combined with a novel anti-calcification treatment makes it a promising substitute in the category of stented bioprostheses. To our knowledge, this is the first study that reports early postoperative outcome and hemodynamic data in a European population.

Procedural data, including cross clamp times, were comparable to other stented bio-prosthetic heart valves and verify the simplicity and safety of the LDP-implantation [4–6]. However, one has to consider the low mean age and predicted risk of the study cohort, which was triggered by the increasing use of transfemoral aortic valve replacement in our institution in older high risk patients. Noticeably, intra-annularly implantation occurred very frequently. This is triggered by the institutional guideline to implant stentless valves in smaller annuli, which leaves the stented valves for larger annuli, where in turn intra-annularly implantation can be advantageous. This proceeding also led to a predominant male study population, by eliminating female patients with small annuli. Consequently, 64% of valve size 27 mm was implanted intra-annularly.

The early clinical results after implantation of the LDP were within normal limits for bioprostheses. The postoperative course of most patients was uneventful. However, the need of permanent pacemakers in six patients was slightly higher than reported for the SJM Trifecta [7]. Moreover, there were eight patients requiring temporary dialysis postoperatively. However, those patients were multi-morbid and all but one had undergone complex procedures. The 30-day mortality of 2.0% was lower than the EuroSCORE II predicted mortality (3.1 ± 3.9%), which is actually one of the best predictors for hospital mortality after aortic valve replacement [8].

At first glance, hemodynamic data of the LDP in this study were conclusive. Mean results, regarding pressure gradients, EOA and EOAI were comparable or even better than other bioprostheses, like the St. Jude Medical Trifecta, the Sorin Mitroflow, the Medtronic Mosaic or the Sorin Freedom Solo [6, 9–11]. While analyzing these data it´s to consider, that the body surface area of our study population was relatively high (but normal and typical for German inhabitants), which lowered the EOAI results. Additionally, only two cases of severe patient-prosthesis-mismatch were evident. These cases occurred in obese patients with valve size 25 mm, where obesity biased (lowered) the EOAI by causing a higher body surface area. Notably, also 8 of the 21 patients with moderate-patient-prosthesis mismatch were obese (body mass index above 30). But at second glance, hemodynamic outcome with regard to the labelled valve sizes showed conflicting results in comparison to various other available bioprostheses. For this evaluation, data of the 21 mm LDP was not considered, due to the low number of cases. Data for valve-sizes 23 mm and 25 mm were comparable to data published for the SJM Trifecta regarding pressure gradients, EOA and EOAI [7, 12]. In contrast, data for size 27 mm showed inferior results than the SJM Trifecta. The comparison to the Sorin Mitroflow, a stented pericardial bioprostheses, showed comparable pressure gradients for valve-sizes 23 mm and 25 mm, whereas LDP size 27 mm showed higher gradients [9]. The EOAI of the Sorin Mitroflow was lower for all valve sizes, but the gap to the LDP was closest for the 27 mm prosthesis. The Medtronic Mosaic, a stented porcine bioprostheses, showed higher mean pressure gradients for valve sizes 23 mm and 25 mm and comparable values for size 27 mm [10]. Upon consideration of the EOA of the Medtronic Mosaic, values were comparable for valve sizes 23 mm and 25 mm, and higher for size 27 mm [10]. The first generation porcine stentless valves (Medtronic Freestyle, SJM Toronto) showed a clear disadvantage in terms of pressure gradients and EOA [13, 14]. On the contrary, the latest generation of pericardial stentless valves showed lower transvalvular gradients compared to our data [11]. Even so, the EOAI of these valves was only slightly above results of the LDP, but once again with the widest gap for valve size 27 mm [11]. According to the comparison with these studies, valve sizes 23 mm and 25 mm showed excellent hemodynamic properties, while a slightly impaired function of valve size 27 mm was evident. Possibly, the high percentage of intra-annularly implanted valves in this size has an impact, due to the change of the hemodynamic flow pattern caused by the stent in the aortic annulus. However, our results showed no difference between the intra-annular and the supra-annular position for valve size 27, possibly due to the low number of cases. Hence, further studies with larger cohorts and a higher number of implants per size are required. Additionally, longer follow-up is necessary to confirm these findings in mid-term and long-term follow-up.

## Conclusion

The Labcor Dokimos Plus was easy to implant, offered operatively no peculiarities and patients showed a satisfactory clinical outcome. Hemodynamic results were pleasing.

## Abbreviations

ECLS: Extracorporeal life support
EOA: Effective aortic valve orifice area
EOAI: Indexed effective aortic valve orifice area
LDP: Labcor Dokimos Plus
